# Opposing Regulation of PROX1 by Interleukin-3 Receptor and NOTCH Directs Differential Host Cell Fate Reprogramming by Kaposi Sarcoma Herpes Virus

**DOI:** 10.1371/journal.ppat.1002770

**Published:** 2012-06-14

**Authors:** Jaehyuk Yoo, Ha Neul Lee, Inho Choi, Dongwon Choi, Hee Kyoung Chung, Kyu Eui Kim, Sunju Lee, Berenice Aguilar, Jinjoo Kang, Eunkyung Park, Yong Suk Lee, Yong-Sun Maeng, Nam Yoon Kim, Chester J. Koh, Young-Kwon Hong

**Affiliations:** 1 Department of Surgery, Department of Biochemistry and Molecular Biology, Norris Comprehensive Cancer Center, Keck School of Medicine, University of Southern California, Los Angeles, California, United States of America; 2 Division of Pediatric Urology, Children's Hospital Los Angeles and University of Southern California Keck School of Medicine, Los Angeles, California, United States of America; University of North Carolina at Chapel Hill, United States of America

## Abstract

Lymphatic endothelial cells (LECs) are differentiated from blood vascular endothelial cells (BECs) during embryogenesis and this physiological cell fate specification is controlled by PROX1, the master regulator for lymphatic development. When Kaposi sarcoma herpes virus (KSHV) infects host cells, it activates the otherwise silenced embryonic endothelial differentiation program and reprograms their cell fates. Interestingly, previous studies demonstrated that KSHV drives BECs to acquire a partial lymphatic phenotype by upregulating PROX1 (forward reprogramming), but stimulates LECs to regain some BEC-signature genes by downregulating PROX1 (reverse reprogramming). Despite the significance of this KSHV-induced bidirectional cell fate reprogramming in KS pathogenesis, its underlying molecular mechanism remains undefined. Here, we report that IL3 receptor alpha (IL3Rα) and NOTCH play integral roles in the host cell type-specific regulation of PROX1 by KSHV. In BECs, KSHV upregulates IL3Rα and phosphorylates STAT5, which binds and activates the PROX1 promoter. In LECs, however, PROX1 was rather downregulated by KSHV-induced NOTCH signal via HEY1, which binds and represses the PROX1 promoter. Moreover, PROX1 was found to be required to maintain HEY1 expression in LECs, establishing a reciprocal regulation between PROX1 and HEY1. Upon co-activation of IL3Rα and NOTCH, PROX1 was upregulated in BECs, but downregulated in LECs. Together, our study provides the molecular mechanism underlying the cell type-specific endothelial fate reprogramming by KSHV.

## Author Summary

Kaposi's sarcoma (KS) is one of the most common neoplasms in HIV-positive individuals and organ transplant recipients. KS-associated herpes virus (KSHV), also known as human herpes virus (HHV)-8, has been identified as the causative agent and infects endothelial cells to form KS. Importantly, we and others have discovered that when KSHV infects endothelial cells of blood vessels, it reprograms host cells to resemble endothelial cells in lymphatic vessels. On the other hand, when KSHV infects endothelial cells in lymphatic vessels, the virus directs the host cells to partially obtain the phenotypes of blood vessel endothelial cells. These host cell reprogramming represent abnormal pathological processes, which are not as complete as the physiological process occurring during embryonic development. Currently, it is not clear how and why this cancer causing virus modifies the fate of its host cells. In this study, we aimed to dissect the molecular mechanism underlying the virus-induced host cell fate reprogramming and found two important cellular signaling pathways, interleukin-3 and Notch, playing key roles in the pathological events. Our current study provides a better understanding of KS tumorigenesis with a potential implication in a new KS therapy.

## Introduction

Kaposi sarcoma (KS) was originally described by a Hungarian doctor Morris Kaposi in 1872, but did not attract much scientific, clinical and public attention until 1980s when acquired immunodeficiency syndrome (AIDS) became an epidemic and KS was subsequently found to be the most common cancer among HIV-positive individuals [1]. KS-associated herpes virus (KSHV), also known as human herpes virus (HHV)-8, was identified in 1994 as the causative agent for KS [2]. KSHV is a member of the gamma herpes virus superfamily and, similar to other herpes virus, has distinct latent and lytic stages. While KS tumor formation is initiated by latent infection of host cells by KSHV, a small fraction of latent cells spontaneously undertakes the lytic phase, a reproductive replication process that releases the infectious viral particles for another round of infection [1]. During the latent infection, KSHV expresses only a handful of genes among its some 90 viral genes and these latent genes include latency-associated nuclear antigen (LANA), viral cyclin-D homolog (vCyc-D), viral FLICE-inhibitory protein (v-FLIP) and kaposin isoforms [3]–[8].

KS tumors are often associated with vessel-like spaces that are filled with immune cells and red blood cells, and the proliferating tumor cells of KS are now believed to be originated from KSHV-infected vascular endothelial cells [9]. The endothelial origin of the KS tumor cells was first proposed about 45 years ago based on the expression of blood vascular endothelial cell (BEC) markers by KS cells [10]. However, identification of new signature genes for lymphatic endothelial cells (LECs) in the 1990s prompted a re-evaluation on the histogenetic origin of the KS tumor cells. A number of research groups have subsequently reported the expression of LEC-markers in all stages of KS tumors, in addition to the previously identified BEC-specific molecules [11]. Moreover, KSHV has been reported to be able to infect both BECs and LECs under *in vitro* culture condition [12].

Importantly, we and other have reported that KSHV induces a host cell fate reprogramming [13]–[15]. When KSHV infects BECs, the virus upregulates a significant number of LEC-signature genes. On the contrary, when KSHV infects LECs, the host cells express some of the BEC-associated genes. Importantly, neither of these host cell fate reprogramming processes is a complete trans-differentiation, but rather KSHV infection directs the host cells to move away from their original cell identities and to end up somewhere in between the two endothelial cell fates, exhibiting mixed cell phenotypes, based on the genome-wide transcriptional profiling studies [12]–[15]. Thus, considering the fact that BECs differentiate to become LECs during embryogenesis [16], KSHV-infected BECs are considered to undertake a “forward” differentiation, and KSHV-infected LECs go through a “reverse” differentiation [12]–[15]. We and others have demonstrated that KSHV induces this host cell type-specific reprogramming by deregulating the expression of PROX1 [12]–[15], a homeodomain transcriptional regulator that functions as the master control protein in LEC-differentiation [16], [17]. Whereas KSHV upregulates PROX1 in BECs where PROX1 is not expressed, the virus downregulates PROX1 in LECs, a cell type which abundantly expresses PROX1 [12]–[15]. This host-specific bidirectional regulation of PROX1 and host cell fate reprogramming by KSHV are of great biological and pathological significance to better understand the host-virus interaction. Nonetheless, the underlying molecular mechanism remains undefined.

Several signal transduction pathways and transcriptional factors have been identified to regulate the expression of PROX1 [18]–[22]. Among them, interleukin (IL)-3 and NOTCH signals have been previously implicated with KS pathogenesis [23]–[26]. The IL-3 receptor (IL3R) consists of a heterodimer of an α-subunit and a β-subunit: While IL3R α-subunit (IL3Rα/CD123) determines the receptor specificity for IL3, IL3R β-subunit (IL3Rβ/CD123), having a long cytoplasmic domain, is responsible for transducing external signals and can form heterodimers with IL5 receptor α-subunit and granulocyte-macrophage colony stimulating factor (GM-CSF) receptor α-subunit to make IL5R and GM-CSFR, respectively [27]. IL-3, a potent stimulator of differentiation of hematopoietic stem cells, was found to trans-differentiate cultured BECs to LECs by upregulating PROX1 expression [18]. Moreover, IL-3 is constitutively expressed and secreted by cultured LECs and required to maintain the LEC-phenotype *in vitro* [18]. On the other hand, we have reported that NOTCH signal suppresses the lymphatic phenotype by downregulating lymphatic cell fate regulators, PROX1 and COUP-TFII, through its effector Hey1 and Hey2 [22].

In this report, we defined the molecular mechanism underlying the host-specific bidirectional regulation of PROX1 by KSHV. We found that KSHV activates both IL3Rα and NOTCH pathways and that these two pathways opposingly regulate PROX1 expression in blood versus lymphatic-lineage endothelial cells. Our data show that IL3Rα pathway serves as an activating signal for PROX1 expression in KSHV-infected BECs and human umbilical vein endothelial cells (HUVECs), whereas NOTCH acts as a repression signal for PROX1 expression in KSHV-infected LECs. In summary, KSHV-induced co-activation of these two opposing signals results in PROX1-upregulation in KSHV-infected BECs and HUVECs, but PROX1-downregulation in KSHV-infected LECs. Together, our data provide the molecular mechanism underlying the cell type-specific regulation of PROX1 by KSHV.

## Results

### KSHV differentially regulates PROX1 expression in blood *vs.* lymphatic-lineage endothelial cells

**Figure 1:**
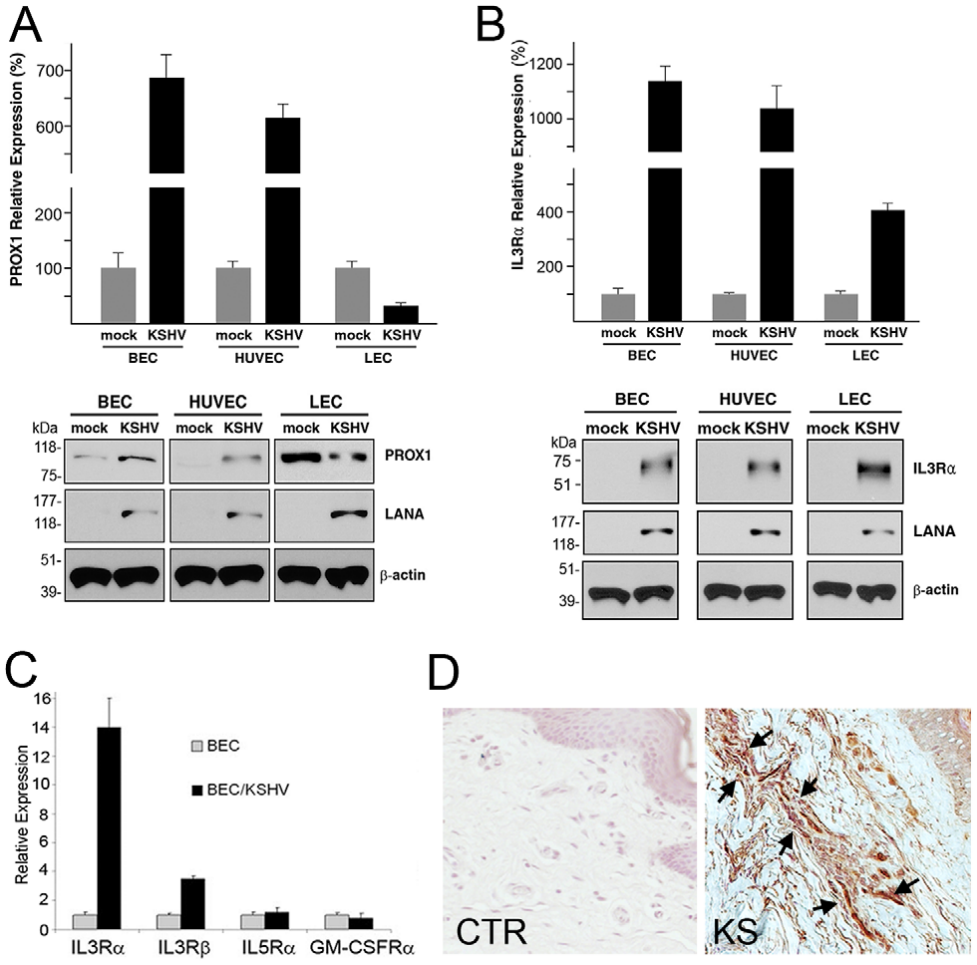
Regulation of the expression of PROX1 and IL3Rα by KSHV in blood vs. lymphatic-lineage endothelial cells. (A) KSHV upregulated PROX1 in BECs and HUVECs, but downregulated PROX1 in LECs based on quantitative real-time RT-PCR (qRT-PCR) and western blot analyses. Latency-associated nuclear antigen (LANA) was used to confirm KSHV-infection and β-actin for equal loading in western analyses. (B) IL3Rα was comparably upregulated by KSHV in the three cell types determined by qRT-PCR and western blot analyses. (C) Expression of IL3Rβ, IL5Rα and GM-CSFRα in BECs by KSHV infection was determined by qRT-PCR. (D) Immunohistochemistry analysis showed prominent expression of IL3Rα in KS tumor cells (arrow-marked) in the skin of a HIV-positive patient. CTR, a control skin section from a normal neonatal foreskin; KS, Kaposi sarcoma tumor section from a HIV-positive individual.

We and others have previously reported that KSHV infection of primary human BECs resulted in upregulation of otherwise silenced PROX1 along with a lymphatic reprogramming of host cell fate [13]–[15]. Interestingly, however, it was also reported that KSHV infection of primary human LECs resulted in downregulation of PROX1 [12]. To investigate whether these seemingly inconsistent results may be due to experimental variations among different research groups, or due to endothelial lineage (BEC vs. LEC)-specific differential regulation of PROX1 by KSHV, we performed a series of comparative analyses of KSHV-mediated PROX1 regulation by using the same donor-derived primary neonatal human BECs and LECs, along with HUVECs from different donors. In agreement with the previous reports [12]–[15], quantitative real time RT-PCR (qRT-PCR) revealed that KSHV-infection upregulated PROX1 in BECs and HUVECs, while downregulating PROX1 in LECs (Fig. 1A). Western blot analyses further confirmed that the steady-state level of PROX1 protein was increased in BECs and HUVECs by KSHV infection, but significantly decreased in LECs upon KSHV-infection. Thus, our study confirmed that KSHV differentially regulates the expression of PROX1 in blood versus lymphatic-lineage endothelial cells by upregulating PROX1 in BECs and HUVECs where PROX1 is not expressed, while downregulating in LECs where PROX1 is abundantly expressed.

### IL3Rα plays an important role in KSHV-mediated PROX1 upregulation in BECs

Previous studies have demonstrated that IL-3 activates PROX1 expression in BECs and HUVECs, but not in LECs, [18] and that KSHV infection of microvascular endothelial cells strongly induces expression and secretion of IL-3 [26]. These reports have led us to investigate whether IL-3 signaling plays a role in PROX1-upregulation by KSHV in BECs and HUVECs. Indeed, KSHV-infection significantly upregulated the expression of IL3 receptor alpha (IL3Rα/CD123) mRNA and protein in BECs, HUVECs and LECs (Fig. 1B). We also determined the expression of related receptors such as IL3Rβ, IL5Rα and GM-CSFRα. While IL3Rβ, the other subunit of IL3 receptor, was found to be upregulated by 4-fold in KSHV-infected BECs, IL5Rα and GM-CSFRα, which can dimerize with IL3Rβ to form receptors for IL5 and GM-CSF, respectively, were not regulated by KSHV (Fig. 1C). Moreover, immunohistochemical analyses of KS tumor sections demonstrated prominent expression of IL3Rα in human dermal KS tumor cells (Fig. 1D).

**Figure 2:**
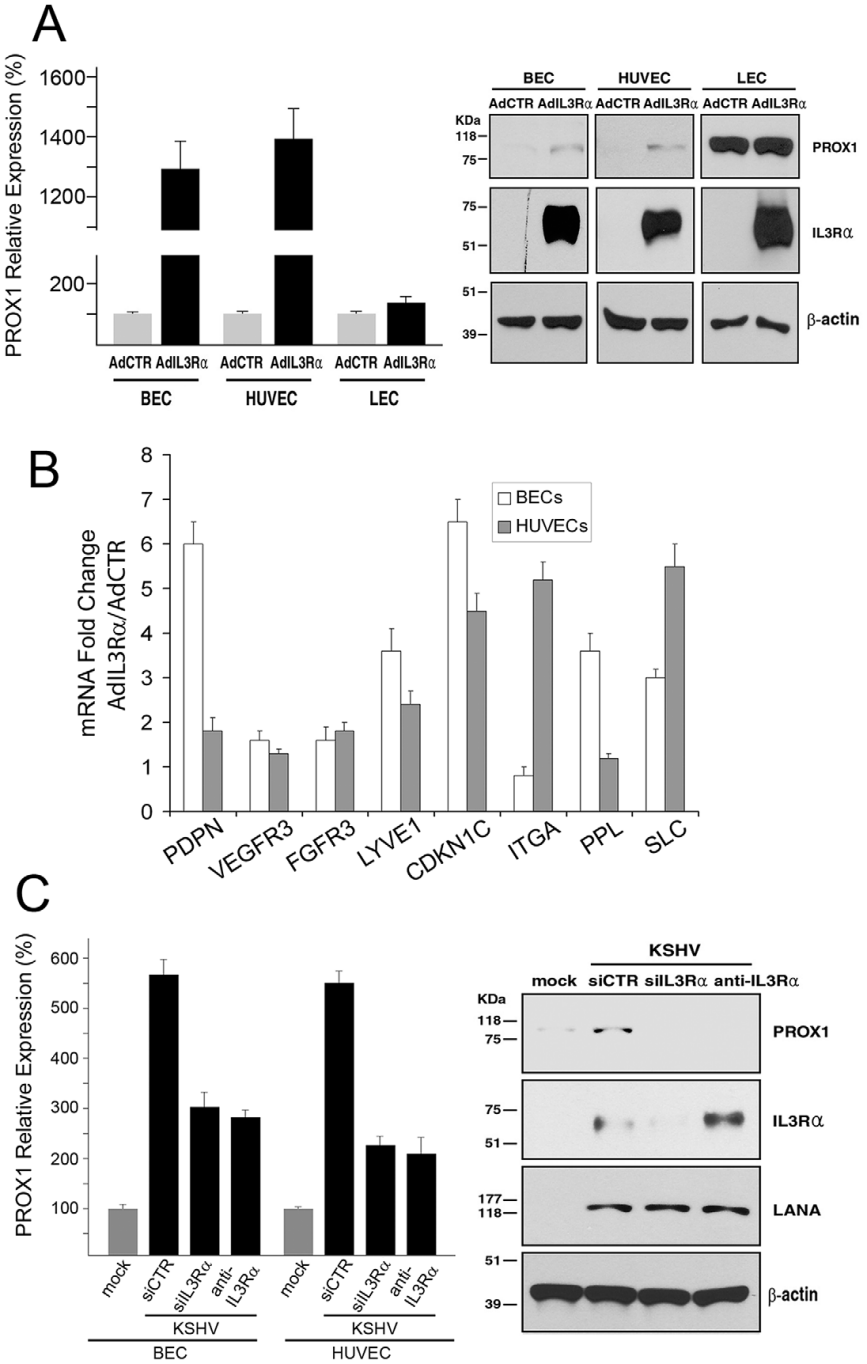
IL3Rα plays a key role in KSHV-mediated PROX1 upregulation. (A) Adenoviral expression of IL3Rα increased the expression of PROX1 mRNA and protein in BECs and HUVECs, but not in LECs based on qRT-PCR and western blot analyses. AdCTR, control adenovirus; AdIL3Rα, IL3Rα-expressing adenovirus. (B) The expression of various LEC-associated genes such as PDPN, VEGFR3, FGFR3, LYVE1, CDKN1C, ITGA1, PPL and SLC was determined by qRT-PCR in BECs or HUVECs transduced by a control (AdCTR) versus IL3Rα- (AdIL3Rα) adenovirus for 48 hours. The graph shows fold changes in expression of each gene by IL3Rα-expressing adenovirus over the control virus. (C) Inhibition of IL3Rα by siRNA or a neutralizing antibody partially abrogated the KSHV-mediated PROX1 upregulation in BECs and HUVECs, as determined by qRT-PCR (BECs and HUVECs) and western blot (BEC only) analyses. siCTR, siRNA for the firefly luciferase; siIL3Rα, siRNA for IL3Rα; anti-IL3Rα, neutralizing antibody against IL3Rα.

Because IL-3 activates PROX1 expression in BECs and HUVECs, but not in LECs [18], we investigated whether overexpression of its receptor, IL3Rα, could upregulate PROX1 in each cell type. We found that adenoviral expression of IL3Rα resulted in a strong upregulation of PROX1 mRNA and protein in BECs and HUVECs, but not in LECs (Fig. 2A), suggesting that the PROX1-upregulating signal by IL3Rα is operative only in PROX1-deficient BECs and HUVECs and does not affect the already abundant expression of PROX1 in LECs. Furthermore, ectopic expression of IL3Rα in BECs and HUVECs resulted in upregulation of various LEC-signature genes such as podoplanin (PDPN), VEGFR3, FGFR3, LYVE1, CDKN1C (p57^Kip2^), ITGA1 (integrin α1), PPL (periplakin) and SLC (secondary lymphoid chemokine) in both BECs and HUVECs (Fig. 2B), indicating that IL3Rα may play an important role in lymphatic reprogramming of KSHV-infected BECs and HUVECs.

We next set out to determine whether IL3Rα is essential for KSHV-mediated PROX1 upregulation in BECs and HUVECs, and thus inhibited IL3Rα using siRNAs or an IL3Rα-neutralizing antibody. Indeed, both approaches showed that PROX1 inhibition could efficiently abrogate the KSHV-induced upregulation of PROX1 mRNA and protein (Fig. 2C). These data demonstrate that IL3Rα plays an integral role in PROX1 upregulation by KSHV in BECs and HUVECs, but not in LECs.

### STAT5 mediates the IL3Rα-induced PROX1 upregulation by directly binding to the PROX1 promoter

**Figure 3:**
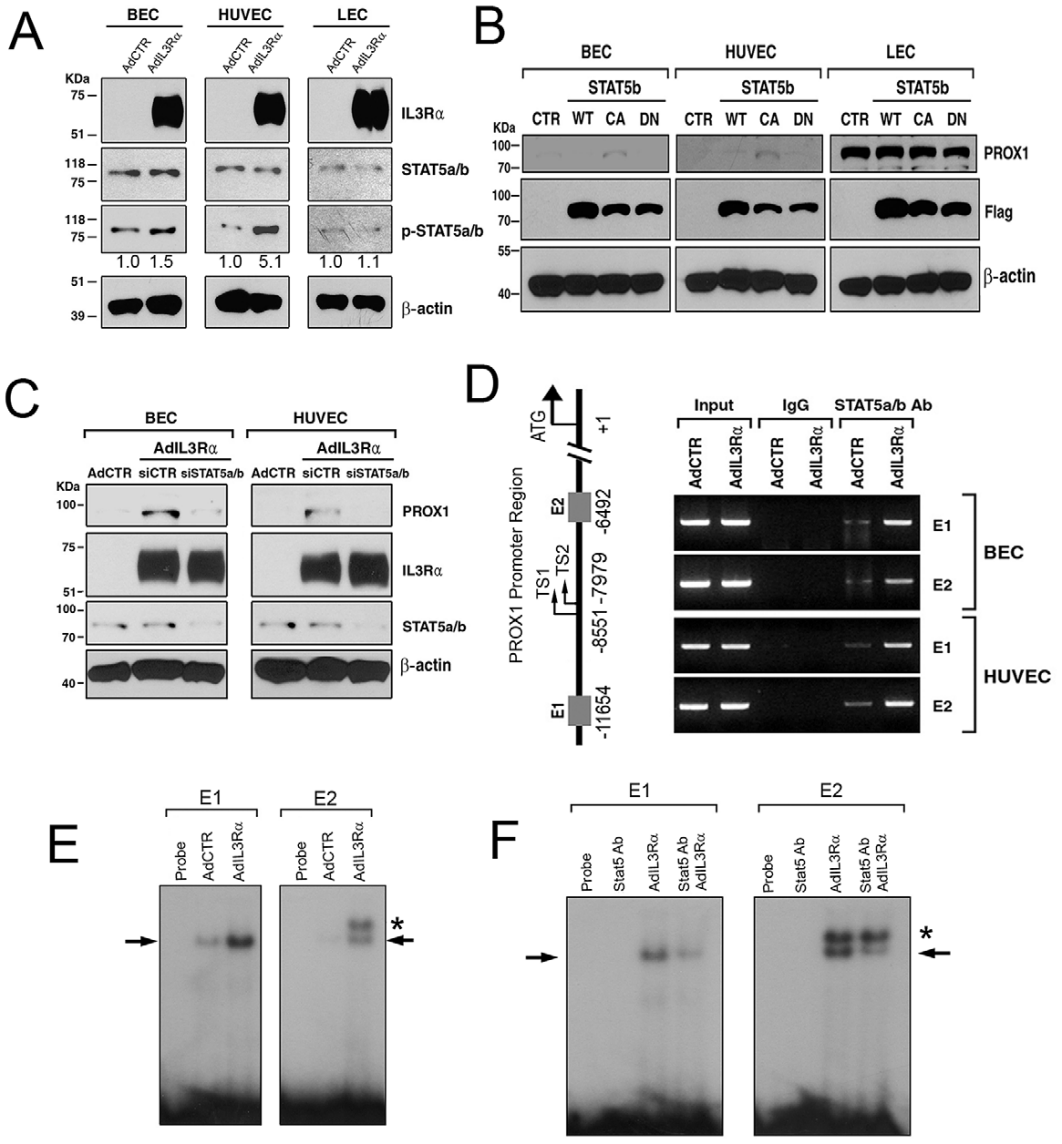
STAT5a/b proteins bind to the PROX1 proximal promoter and mediate IL3Rα-induced PROX1 upregulation. (A) Adenoviral overexpression of IL3Rα increased phosphorylation of STAT5a/b (p-STAT5a/b) in BECs and HUVECs, but not LECs. AdCTR, control adenovirus; AdIL3Rα, IL3Rα-expressing adenovirus. The relative intensity ratio of the p-STAT5a/b band over the total STAT5a/b band is shown below the p-STAT5a/b western blot result. (B) The constitutively active (CA), but not wild type (WT) or dominant negative (DN), form of STAT5b induced PROX1 upregulation in BECs and HUVECs, but not in LECs. Comparable expression of FLAG-tagged rat STAT5b proteins (Flag) was confirmed by western blot assays. CTR, a control vector. (C) Inhibition of STAT5a/b with siRNA-mediated knockdown abrogated the IL3Rα-induced PROX1 upregulation in BECs and HUVECs. siCTR, siRNA for luciferase gene; siSTAT5a/b, siRNA for STAT5a/b. (D) STAT5a/b ChIP assay against the PROX1 promoter sequences revealed an increased binding of STAT5a/b proteins to the two newly identified putative STAT5 binding sites (E1 and E2) upon adenoviral IL3Rα-overexpression in BECs and HUVECs. Relative locations of E1, two PROX1 transcriptional start sites (TS1 based on the PROX1 Ensemble exon number ENSE00001936642 and TS2 based on ENSE00001443122), E2 and the PROX1 initiation codon (ATG) are illustrated. (E) EMSA showing that nuclear fractions isolated from BECs contain binding activity for the E1 and E2 sites and that this binding activity was increased upon IL3Rα-overexpression. (F) The binding activity to the E1 and E2 sites could be inhibited by an anti-STAT5a/b antibody, indicating that the binding activity was caused by STAT5a/b proteins. Arrow-pointed bands in E and F indicate STAT5a/b protein complex with the isotope-labeled E1 and E2 DNA probes. Asterisks point a protein/probe complex that did not respond to the anti-STAT5a/b antibody and thus was likely caused by an unrelated factor.

The key downstream mediators for the IL3/IL3Rα pathway include Jak2 and STAT5a/b, and activated STAT5a/b proteins rapidly enter the nuclei and bind to the promoters of target genes to modulate their gene expressions [28]. We thus investigated whether STAT5a/b proteins are involved in the IL3Rα-mediated PROX1 upregulation. Adenoviral overexpression of IL3Rα in BECs, HUVECs and LECs revealed an increased phosphorylation in STAT5a/b in BECs and HUVECs, but at much lesser degree in LECs (Fig. 3A). We then overexpressed the wild type, constitutive active or dominant negative form of STAT5b protein in BECs, HUVECs and LECs by transient transfection and determined their effects on PROX1 expression. Interestingly, the ectopic expression of the constitutive active form of STAT5b protein resulted in upregulation of PROX1 protein in BECs and HUVECs, but did not change PROX1 expression in LECs (Fig. 3B). Moreover, the IL3Rα-induced PROX1 upregulation was significantly abrogated when the expression of STAT5a/b was inhibited by siRNA-mediated knockdown (Fig. 3C), establishing a key role of STAT5a/b in the IL3Rα-induced PROX1 upregulation.

To further corroborate the molecular interaction between STAT5a/b protein and the PROX1 gene, we next searched for putative binding sites of STAT5a/b proteins in the PROX1 promoter regions of different animal species and mapped two or three candidate sites in the PROX1 promoter regions of nine mammals (human, chimp, mouse, rat, guinea pig, horse, rabbit, dog and marmoset) (Supplemental Fig. S1). Importantly, both DNA sequences and relative locations of the putative STAT5a/b binding sites were found to be highly conserved among the nine mammalian species. To further validate them as STAT5a/b binding sites, we next performed chromatin immunoprecipitation (ChIP) and gel electrophoretic mobility shift assays (EMSA) for the human PROX1 promoter region. Indeed, our ChIP assay detected a basal binding activity of endogenous STAT5 proteins to the two putative STAT5a/b sites (termed E1 and E2) found approximately 11.7- and 6.5-kb upstream, respectively, from the human PROX1 initiation codon in BECs and HUVECs, and these binding activities were significantly increased by IL3Rα overexpression (Fig. 3D). Moreover, EMSA demonstrated that nuclear extracts isolated from IL3Rα-overexpressed BECs efficiently caused a shift in the mobility of both E1 and E2 probes (Fig. 3E). Addition of an anti-STAT5a/b antibody in the EMSA reactions significantly inhibited formation of the protein/probe complexes (Fig. 3F), indicating that E1 and E2 probes made DNA/protein complexes with STAT5a/b proteins. Taken together, these data demonstrate that STAT5a/b proteins play a significant role in the IL3Rα-induced PROX1 upregulation in BECs and HUVECs by directly binding to the PROX1 promoter region.

### NOTCH directs the KSHV-mediated PROX1 downregulation in lymphatic endothelial cells

**Figure 4:**
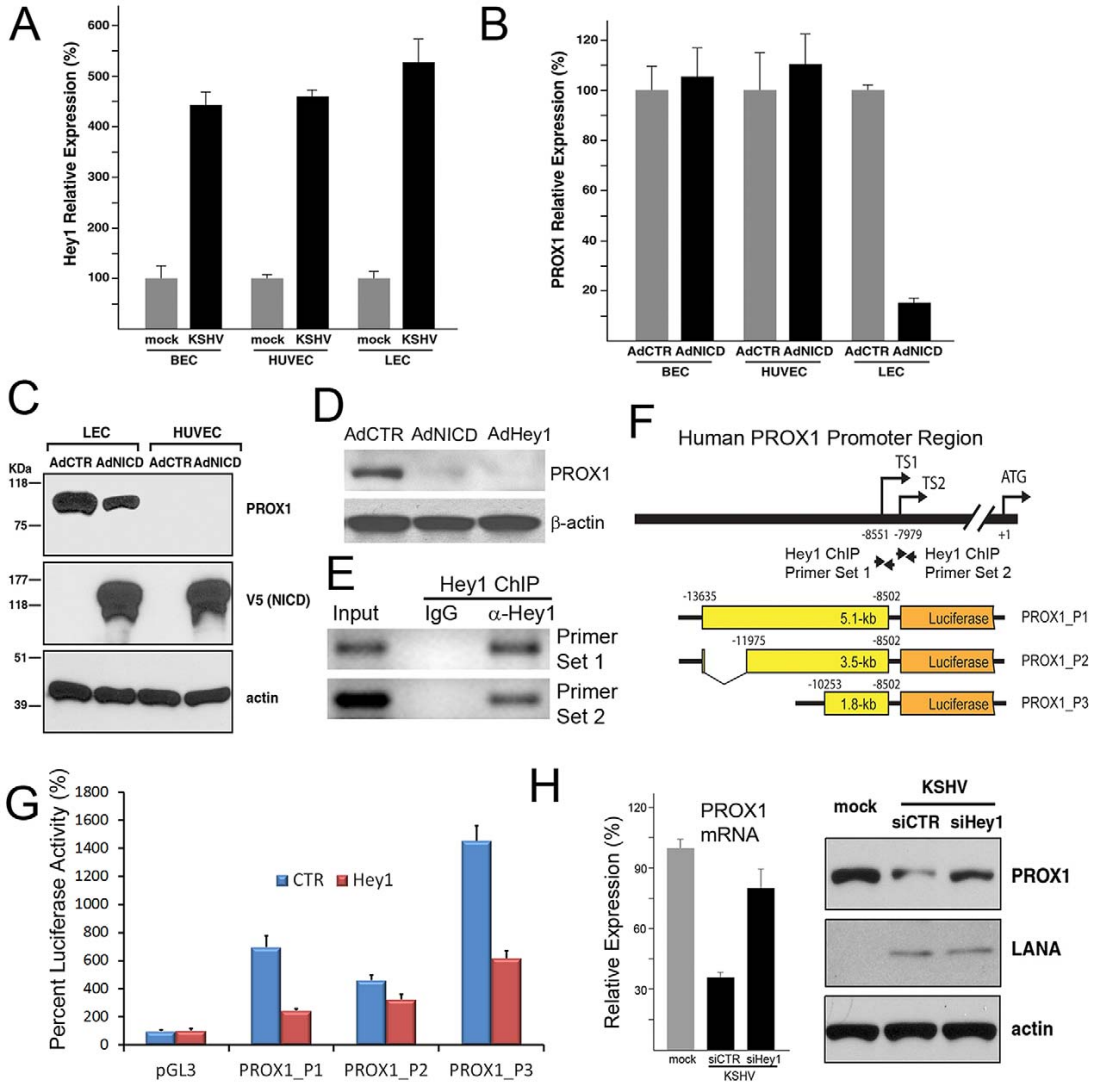
NOTCH pathway plays a major role in KSHV-mediated PROX1 downregulation in LECs. (A) HEY1, an effector of NOTCH, was strongly upregulated by KSHV in BECs, HUVECs and LECs, as determined by qRT-PCR. (B,C) While activated NOTCH repressed PROX1 expression in LECs, the PROX1 expression level remained unchanged in BECs or HUVECs by NOTCH, based on qRT-PCR (B) and western blot (C) analyses. AdCTR, control adenovirus; AdNICD, NICD-expressing adenovirus. (D) Expression of PROX1 protein was strongly repressed by adenoviral overexpression of NICD and HEY1 in LECs based on western blot analyses. AdHey1: FLAG-HEY1-expressing adenovirus. (E) HEY1 ChIP assay against the human PROX1 promoter shows that HEY1 protein is associated with the PROX1 promoter. Primary LECs transduced with AdHey1 (expressing FLAG-tagged HEY1) were used to perform ChIP assays using an anti-FLAG antibody and two sets of primers against the human PROX1 promoter. Relative location of the primer sets is marked in panel F. (F) A diagram illustrating the relative location of the start codon (ATG), two PROX1 transcription start sites (TS1, TS2), the HEY1 ChIP primer sets and the boundaries of the PROX1 promoter fragments that were used to generate the luciferase reporter constructs. (G) Luciferase-reporter assays using PROX1-promoter constructs revealed that PROX1 repression by HEY1 can be mediated through a 1.8-kb promoter fragment (PROX1_P3). (H) Inhibition of HEY1 by siRNA abrogated the KSHV-mediated downregulation of PROX1 based on qRT-PCR and western blot analyses.

We next set out to investigate how KSHV-infection resulted in downregulation of PROX1 in LECs, despite the fact that KSHV upregulates PROX1 in BECs and HUVECs. Previous studies have shown an increased activity of the NOTCH pathway in KSHV-infected endothelial cells and KS-tumor cells *in vivo* [23], [25], [29], [30]. Moreover, we have recently reported that activated NOTCH represses PROX1 expression through HEY1 in LECs [22]. Accordingly, we came up with a hypothesis that KSHV-induced NOTCH activation may be involved in PROX1 downregulation in KSHV-infected LECs. Supporting this hypothesis, KSHV-infection of all three cell types, BECs, HUVECs and LECs, resulted in upregulation of HEY1 (Fig. 4A). Adenoviral overexpression of NOTCH intracellular domain (NICD) caused a significant downregulation of PROX1 in LECs, but not in BECs and HUVECs (Fig. 4B&C). In addition, Notch activation in LECs resulted in downregulation of additional lymphatic-signature genes such as podoplanin (PDPN) and CDKN1C, suggesting a suppressive role of Notch signaling in LEC phenotypes (Supplemental Fig. S2). Moreover, microarray-based analyses on the NICD-induced modulation of the transcriptional profiles in primary LECs (National Center for Biotechnology Information, Gene Expression Omnibus accession number: GSE20978) support the effect of Notch on LEC phenotype. Furthermore, we found that HEY1, like NICD, was able to strongly repress the expression of PROX1 protein, when overexpressed in LECs (Fig. 4D). Since HEY1 is known to repress target gene expression by binding to the promoter [31], we performed HEY1-ChIP assays against the PROX1 promoter in primary LECs and found that HEY1 was indeed physically associated with the PROX1 promoter around the two transcriptional start sites (Fig. 4E&F). We then generated a set of PROX1-promoter reporter constructs and found that a 1.8-kb proximal promoter region was sufficient to deliver the HEY1-mediated repression (Fig. 4G). Finally, inhibition of HEY1 expression by siRNA abrogated the KSHV-mediated downregulation of PROX1 mRNA and protein in LECs (Fig. 4H). Together, these findings demonstrate that NOTCH activation is responsible for the KSHV-mediated PROX1 downregulation in LECs, but not in BECs and HUVECs, and that the NOTCH effector HEY1 directly binds to the PROX1 promoter to downregulate its gene expression.

### PROX1 is required to maintain the expression of HEY1 in LECs: Reciprocal regulation between PROX1 and HEY1

**Figure 5:**
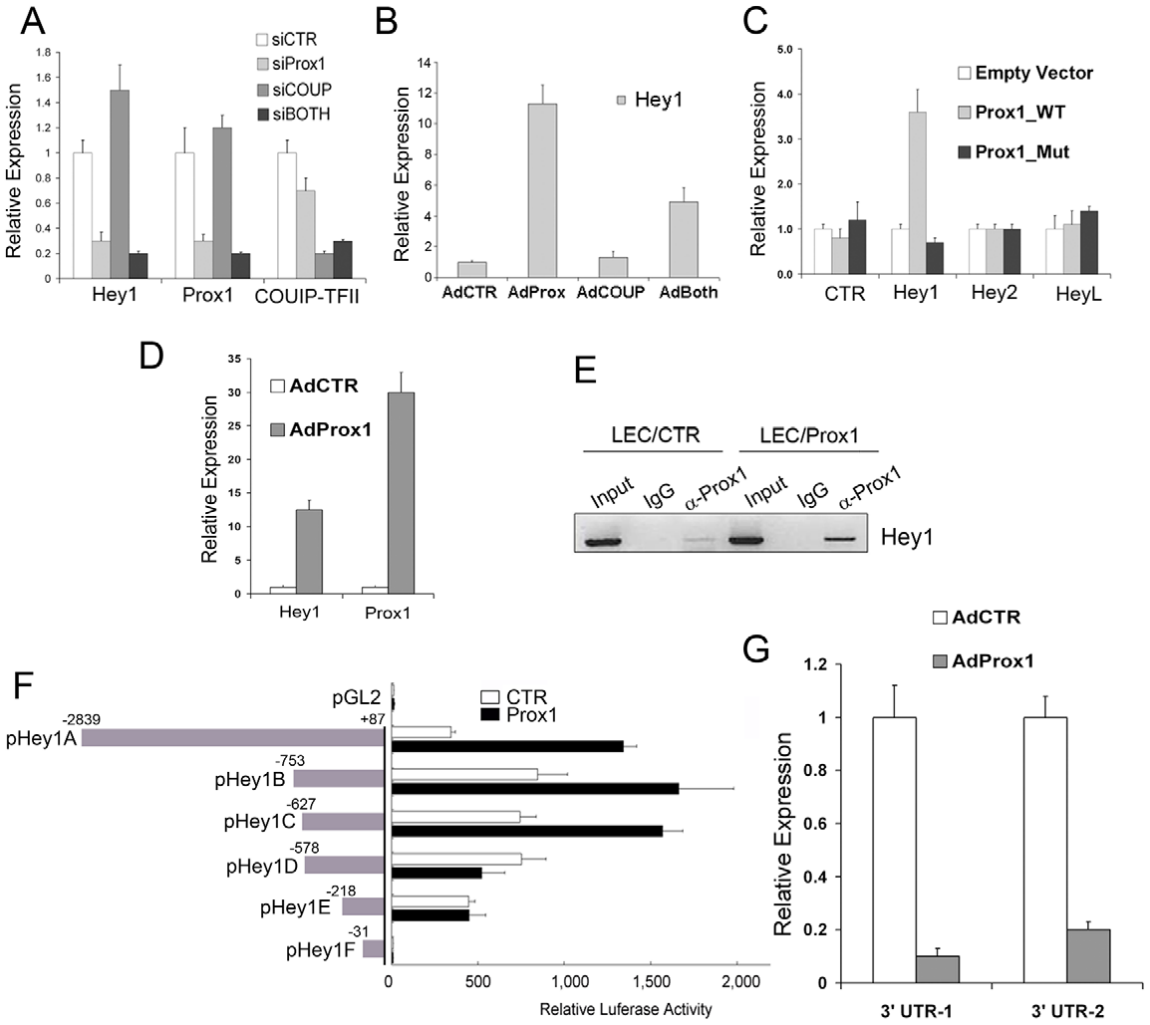
PROX1 upregulates HEY1 in primary LECs. (A) Knockdown of PROX1, but not COUP-TFII, resulted in downregulation of HEY1 in LECs. qRT-PCR analyses were performed to determine the expression of HEY1, PROX1 and COUP-TFII in primary LECs that were transfected for 48 hours with siRNAs targeting the firefly luciferase (siCTR), PROX1 (siProx1), COUP-TFII (siCOUP) or both PROX1 and COUP-TFII (siBOTH). (B) Adenoviral expression of PROX1, but not of COUP-TFII, increased the expression of HEY1 mRNA in LECs. qRT-PCR analyses were performed to determine the level of HEY1 mRNA in LECs that were transduced with a control (AdCTR), PROX1 (AdProx1), COUP-TFII (AdCOUP) adenovirus or with both PROX1 and COUP-TFII (AdBOTH) for 48 hours. (C) Luciferase reporter constructs of the promoters of HEY1, Hey2 or HeyL were transiently transfected into HEK293 cells along with a control vector (Empty Vector), PROX1 wild type (Prox1_WT) or PROX1 DNA-binding mutant (Prox1_Mut). After 48 hours, luciferase activity was measured and each value was normalized by total cell lysate amounts. (D) PROX1 also upregulated HEY1 in aortic endothelial cells (human umbilical aortic endothelial cells, HUAEC). Relative expression was determined for HEY1, Hey2 and PROX1 in primary HUAECs that were transduced with a control or PROX1-adenovirus for 48 hours. (E) Chromatin immunoprecipitation (ChIP) assay demonstrating a physical association between PROX1 protein and the HEY1 promoter in LECs. Primary LECs were transfected with a control (LEC/CTR) or a PROX1-expressing vector (LEC/Prox1) for 48 hours and then subjected to ChIP analyses using a normal IgG (IgG) or anti-PROX1 (α-Prox1) antibodies. (F) A ∼0.6-kb HEY1 proximal promoter was sufficient to deliver the PROX1-mediated repression of HEY1 expression. Reporter constructs of the HEY1 promoter were transfected with a control or PROX1-expressing vector into HEK293 cells for 48 hours. (G) Ectopic expression of PROX1 in LECs resulted in downregulation of endogenous PROX1. LECs were transduced with a control (AdCTR) or PROX1 (AdProx1) adenovirus. After 48 hours, qRT-PCR was performed by using two sets of probes that target the 3′ untranslated region (UTR) of endogenous PROX1. All data were shown as a relative average expression ± standard deviation (SD).

We have previously reported that PROX1 physically and functionally interacts with the orphan nuclear receptor COUP-TFII to specify the cell fate of LECs [32]. We also performed a genome-wide search for PROX1 target genes using microarray analyses and identified a list of genes, whose expression was altered by PROX1 knockdown in LECs [32]. Interestingly, the microarray analyses revealed that the expression of HEY1 was significantly downregulated in LECs by PROX1 knockdown (GEO accession: GSE12846). This unexpected regulation of HEY1 by PROX1 was further confirmed using qRT-PCR of total RNAs isolated from LECs that were transfected with siRNA against PROX1 and/or COUP-TFII (Fig. 5A). Notably, knockdown of COUP-TFII, a PROX1-interacting protein, did not alter the HEY1 expression. On the contrary, adenoviral overexpression of PROX1, but not COUP-TFII, in LECs resulted in a strong upregulation of HEY1 (Fig. 5B). We then asked whether PROX1 could activate the proximal promoter of HEY1 and thus performed a series of luciferase reporter assays using promoter constructs of HEY1 and two other HEY family members, HEY2 and HEYL. Indeed, the HEY1 promoter was found to be activated by PROX1 wild type, but not by a PROX1 mutant lacking DNA-binding activity [33] (Fig. 5C). Moreover, adenoviral expression of PROX1 in human umbilical aortic endothelial cells (HUAEC) also resulted in upregulation of HEY1 (Fig. 5D). We then performed PROX1 ChIP assays against the HEY1 promoter and found that PROX1 protein is physically associated with the HEY1 promoter (Fig. 5E). Subsequently, a set of HEY1 promoter reporter constructs was generated and used to further study the PROX1 regulation of HEY1. Notably, a ∼0.7 kb-long HEY1 promoter (pHey1C) was sufficient to deliver the PROX1-mediated activation of the HEY1 promoter (Fig. 5F). Therefore, we concluded that PROX1 positively regulates the expression of HEY1 by directly binding to its promoter. Together with the findings above (Fig. 4), these data established a reciprocal regulation between PROX1 and HEY1: HEY1 functions as a repressor of PROX1 and PROX1 is required to upregulate or maintain HEY1 expression. According to this reciprocal feedback regulation, PROX1 could negatively regulate its own gene expression. To confirm this auto-regulation, we ectopically overexpressed PROX1 in LECs using adenovirus that harbors the PROX1 open reading frame (ORF) only and then determined the expression level of the endogenous PROX1 by two qRT-PCR probes detecting the PROX1 3′-untranslated region (UTR), which is not present in the adenovirus. Indeed, ectopic expression of PROX1 resulted in a significant downregulation of the endogenous PROX1 (Fig. 5G). Taken together, our data uncovered an intricate auto-regulatory mechanism for the PROX1 gene expression that utilizes the HEY1 repressor, a component of NOTCH signal pathway.

### Differential expression of PROX1 by two opposing regulatory forces in blood versus lymphatic-lineage endothelial cells

**Figure 6:**
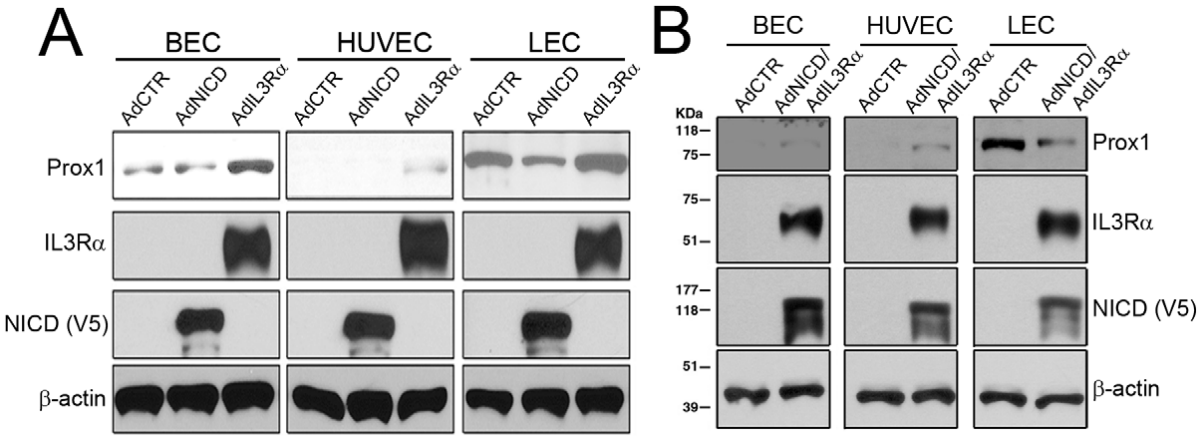
IL3Rα and NOTCH pathways opposingly regulate PROX1 and bring up different outcomes in BECs/HUVECs versus LECs. (A) Over-expression of NICD in BECs and HUVECs did not significantly alter PROX1 expression in BECs and HUVECs, but downregulated PROX1 in LECs. In comparison, expression of IL3Rα induced PROX1 expression in BECs and HUVECs, but did not further upregulate PROX1 in LECs. (B) Simultaneous expression of NICD and IL3Rα resulted in PROX1 upregulation in BECs and HUVECs, but PROX1 downregulation in LECs. NICD is tagged with the V5 antigen.

Since our studies above showed that KSHV activates both IL3Rα and NOTCH pathways simultaneously, we next asked how these two signals counteract with each other in regulating the expression of PROX1 in BECs versus LECs. To address this question, we concurrently activated both pathways by adenoviral expression of IL3Rα and NICD in BECs, HUVECs and LECs. Notably, co-expression of IL3Rα and NICD resulted in differential regulation of the PROX1 expression in blood vs. lymphatic-lineage endothelial cells (Fig. 6A,B). In BECs and HUVECs, PROX1 was found to be upregulated by co-expression of IL3Rα and NICD. In LECs, however, PROX1 was rather downregulated by activation of the two pathways. These data indicate that the IL3Rα-induced PROX1 activating signal is more effective than the NICD-mediated PROX1 repression in BECs and HUVEC, resulting in PROX1 upregulation. On the contrary, the NICD-mediated repression is more prominent than the IL3Rα-induced activation in LECs, causing downregulation of PROX1. Taken together, co-activation of the IL3Rα and NOTCH pathways yields a differential expression of PROX1 and may account for the KSHV-mediated endothelial lineage-specific differential regulation of PROX1 and accompanying host cell fate reprogramming.

## Discussion

Despite their morphological and functional similarities, endothelial cells exhibit remarkable heterogeneity and plasticity. Heterogeneity of endothelial cells is profoundly contributed by their plastic cell fates in response to internal and external stimuli such as immunological, functional, metabolic, anatomical and hemodynamic signals [34], [35]. In addition to these physiological stimuli, pathological insults can also incite the plasticity of endothelial cell identities. We and others have previously reported that KSHV-infection reprograms the host cell identity by triggering a drift from their original phenotypes and that PROX1, whose expression is deregulated by KSHV, plays a key role in this pathological host cell fate reprogramming [12]–[15]. Although this concept of the pathogen-induced host cell fate reprogramming has forwarded a new view on the histogenetic origin of KS tumor cells [12]–[15], two subsequent questions, *how* and *why*, remained to be answered. In this study, we aimed to address the first question, *how*, by studying the KSHV-mediated regulation of PROX1 in context of the virus-induced host cell fate plasticity.

**Figure 7:**
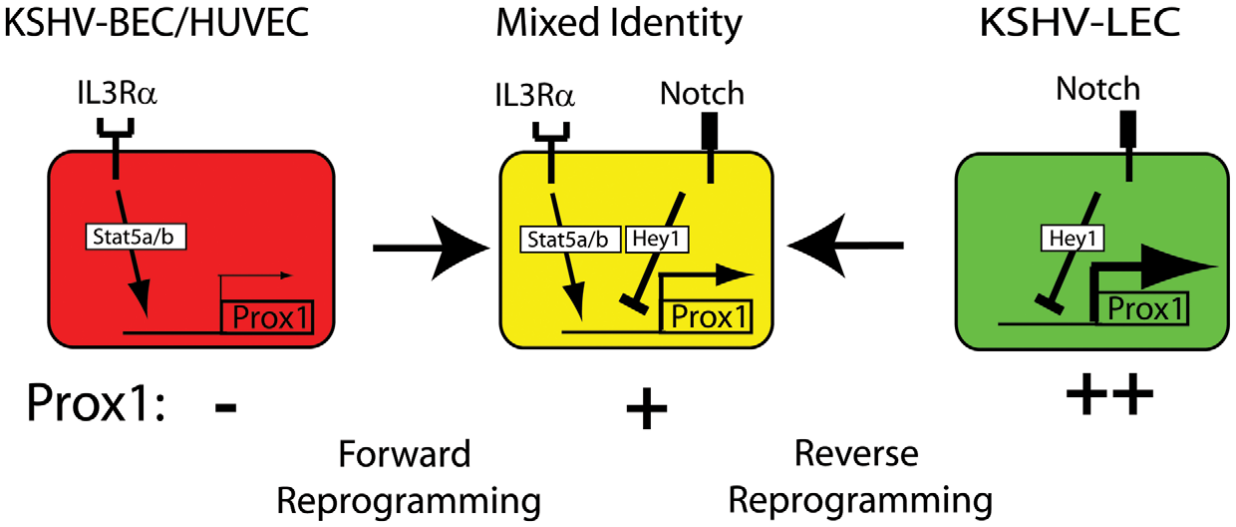
Working hypotheses for the KSHV-mediated bidirectional host cell fate reprogramming and for the opposing regulation of PROX1 by IL3Rα and NOTCH. PROX1 expression level of host cells is the key determinant for the differential PROX1 regulation by KSHV. In PROX1-absent BECs and HUVECs, the PROX1-inducing IL3Rα pathway overwhelms the PROX1-repressive NOTCH pathway, resulting in PROX1 upregulation and acquisition of LEC-phenotype (Forward Reprograming). In PROX1-abundant LECs, the PROX1-repressive NOTCH pathway is more productive than the PROX1-inducing IL3Rα pathway, resulting in PROX1 downregulation and regaining BEC-phenotype (Reverse Reprograming). Importantly, however, these reprogramming processes appear to be incomplete differentiation events and thus KSHV-infected endothelial cells exhibit both (mixed) BEC and LEC-phenotypes.

By using primary BECs and LECs from the same donors, we confirmed that KSHV-infection induces endothelial cell type-specific differential regulation PROX1, as previously reported [13]–[15]. Because only unidirectional differentiation (BECs to LECs) occurs during physiological (embryonic) endothelial cell differentiation, it is important to understand how KSHV pathologically activates a differentiation program by upregulating PROX1 in one cell type and a de-differentiation by downregulating PROX1 in another cell type [12]. Based on the data presented above, we propose a novel hypothesis for the molecular mechanism underlying the KSHV-mediated host cell type-specific regulation of PROX1 (Fig. 7A). We hypothesize that KSHV-infection results in simultaneous activation of the IL3Rα and NOTCH pathways and delivers both positive and negative regulatory signals, respectively, to the *PROX1* gene in both blood and lymphatic-lineage endothelial cells. Under this condition, we believe that it is the initial expression status of PROX1 that brings up the differential consequences: An activating signal (IL3Rα) will cause a discrete change than a repressive signal (NOTCH) in the PROX1-deficient BECs and HUVECs, whereas a repressive signal (NOTCH) will have a higher impact than an activating signal (IL3Rα) in the PROX1-expressing LECs. Moreover, because LECs constitutively secret IL-3 to maintain their phenotypes [18], IL3Rα-mediated signaling is already active in LECs and thus the increased expression of IL3Rα by KSHV does not provide an additional upregulation of PROX1. Accordingly, KSHV-mediated upregulation of PROX1 directs a “forward” reprogramming (differentiation) of BECs and HUVECs to acquire the lymphatic phenotype, but KSHV-mediated downregulation of PROX1 enables a “reverse” reprogramming (dedifferentiation) of LECs. Importantly, since both pathological cell fate reprogramming are incomplete processes, KSHV-infection forces host endothelial cells to move away from their original cell fates and to end up somewhere in between the two endothelial cell fates, as described by previous studies [12]–[15].

Another key finding in the current study is the reciprocal regulation between PROX1 and HEY1 (Fig. 7B). We found that HEY1, the only transcriptional repressor of PROX1 identified to date [22], directly binds to the PROX1 proximal promoter to repress its transcription. Moreover, our current study revealed that PROX1 is required to maintain the expression of HEY1 in LECs. This PROX1/HEY1 reciprocal regulation put forward three important implications. First, a positive regulatory signal of PROX1 expression will be always counteracted by the parallel upregulation of its own repressor HEY1 and thus PROX1 expression will be maintained under a certain threshold level that is set by HEY1. This speculation is consistent with the finding that the activating signal by IL3Rα did not additionally increase PROX1 expression in LECs where PROX1 is already abundantly expressed (Fig. 2A). Second, since HEY1 has been known to repress the expression of the viral lytic phase initiator gene RTA and thus to play an important role in the latency control [36], [37], PROX1 may indirectly contribute to host regulation of the viral latency phase by maintaining the HEY1 expression. Third, HEY1 requires PROX1 for its own expression and thus will be unable to entirely shut down PROX1 expression, even if NOTCH signal is activated. This regulation will serve as a feedback control to counteract the NOTCH-induced repression of PROX1, which is mediated by HEY1. Therefore, the reciprocal regulation between PROX1 and HEY1 adds another layer of complexity to the opposing regulatory circuits of PROX1 expression by IL3Rα and NOTCH upon KSHV-infection.

Moreover, since KS cells secrete a number of chemokines and cytokines and also recruit various immune cells, multiple signal transduction pathways have been associated with KS tumorigenesis [8], [9]. Considering the numerous chemokines and cytokines that KS cells are constantly exposed to [26], [38], [39], it is likely that PROX1 expression would be controlled by multiple activating and repressing signals. In fact, activation of Jak2/Stat3 by gp130 has been demonstrated to be important in KSHV-mediated upregulation of PROX1 and lymphatic reprogramming [40]. Gp130/Stat3 and IL3Rα/STAT5 may cooperate to regulate the PROX1 expression in KSHV-infected cells, especially when the two pathway are known to cross-talk with each other [41]. Supporting this notion, inhibition of IL3Rα in KSHV-infected BECs and HUVECs only partially abrogated PROX1 upregulation by KSHV (Fig. 2C).

One important question brought up by the current study is to determine which KSHV viral proteins are responsible for the upregulation of IL3Rα and NOTCH. We speculate that the latent protein kaposin-B may be involved in upregulation of IL3Rα because kaposin-B has been reported to upregulate expression of various cytokines such as granulocyte-macrophage colony-stimulating factor (GM-CSF) via stabilizing their mRNAs [42] and, notably, GM-CSF has been demonstrated to be a key activator of IL3Rα expression in different cell types [43]–[46]. Therefore, it will be very interesting to investigate if kaposin-B regulates IL3Rα expression. On the other hand, the molecular mechanism underlying the KSHV-mediated NOTCH upregulation has been recently documented by two studies [25], [47], which identified the KSHV-encoded vFLIP, LANA and vGPCR to be responsible for the upregulation of various NOTCH signal components, including the receptors (NOTCH1∼4), ligands (Dll1/4, Jag1) and downstream effectors (Hey1) of the NOTCH signaling pathway.

Although our current study addresses the *how* part to a certain extent, numerous questions on *why* remain unanswered: *Why does KSHV induces its host cell fate reprogramming?* It will be intriguing to determine whether this host cell fate reprogramming is a mere by-product of the viral infection of endothelium, a cell type that happens to be highly plastic in their cell identity, or whether the compromised cell identity provides any pathological advantage to KS tumorigenesis. Further studies should be warranted on these important questions.

## Materials and Methods

### Cell cultures and gene expression reagents

Human primary dermal blood vascular endothelial cells (BECs) and lymphatic endothelial cells (LECs) were isolated from de-identified neonatal human foreskins and cultured as previously described [32] with an approval of the University of Southern California Internal Review Board (PI: YK Hong). Primary human umbilical venous endothelial cells (HUVECs) and human umbilical aortic endothelial cells (HUAEC) were purchased from Lonza (Basel, Switzerland) and cultured in EGM-2 medium (Lonza). Primary endothelial cells were transfected with siRNA by electroporation as previously described [48]. Sequence information of siRNAs are as follows: human HEY1 [22], PROX1 [22], STAT5a/b (SC29495, Santa Cruz Biotechnology), COUP-TFII (UCGUACCUGUCCGGAUAUA, UAUAUCCGGACAGGUACGA), control (fire fly luciferase, CUUACGCUGAGUACUUCGAdTdT), and IL3Rα (CUGGGACCUUAACAGAAAUdTdT). Adenovirus for PROX1 [49], COUP-TFII [32] and NICD [50] were previously described. Adenovirus expressing V5-tagged NICD was a kind gift from Dr. Lucy Liaw (Maine Medical Center Research Institute) [51]. Adenovirus for human IL3Rα was constructed by transferring the IL3Rα coding sequences from AxCALNLhIL-3Rα (Riken BRC DAN Bank, Japan) into Ad-Track-CMV shuttle vector and then recombined with AdEasy-1 based on the reported protocol of adenovirus construction [52]. Expression vectors for Flag-tagged rat-STAT5b (wild type, constitutive active, dominant negative) [53] were kind gifts from Dr. Peter Rotwein (Oregon Health and Science University).

### KS specimen, KSHV production and infection

KS specimens were provided from the AIDS and Cancer Specimen Resource (ACSR) with an approval of the University of Southern California Internal Review Board (PI: YK Hong). Infectious KSHV was purified from BCBL-1 cells as we previously described [54]. Endothelial cells were infected with KSHV for 2∼4 days and infectivity was measured by western blot analyses for KSHV LANA. For inhibition of IL3Rα, BECs or HUVECs were transfected with IL3Rα siRNA or treated with anti-IL3Rα antibody (1 µg/ml) 24 hours before KSHV-infection and 48 hours post infection, RNA and whole cell lysates were harvested for further analyses. Blocking of STAT5a/b expression by siRNA was similarly performed.

### Quantitative real time RT-PCR, immunostaining and immunoblot analyses

Real-time RT-PCR was performed by using TaqMan EZ RT-PCR Core Reagent (Applied Biosystems). Each reaction was multiplexed for target gene and β-actin for normalization. Sequences of two Taqman primer/probes for the 3′ UTR are (TGGTTTTCCCTTTTACAATCGAA/GAATTTGGAGAGACAGGCTTTTG/FAM-TTGTGCCTCCCAAGTGCATTGGAA-TAMRA; TGGTTTTCCCTTTTACAATCGAA/GAATTTGGAGAGACAGGCTTTTG/FAM-TTGTGCCTCCCAAGTGCATTGGAA-TAMRA). Sequences for other primers and probes used for this study will be provided upon request. Immunostaining was performed on formalin-fixed paraffin embedded KS tissue specimens by following a standard immunostaining protocol [32]. Sources of antibodies for immunostaining or western blot analyses are PROX1 (Millipore Corporation, MA), β-actin and Flag (Sigma-Aldrich Corporation), STAT5 and phospho-STAT5 (Cell Signaling Technology), IL3Rα (clone 7G3, BD Bioscience), V5 (Invitrogen) and LANA (Advanced Biotechnologies Inc, Maryland).

### ChIP and EMSA

ChIP assays were performed as previously described [22]. BECs, HUVECs or LECs transduced with a control or IL3Rα-expressing adenovirus for 48 hours were subjected to ChIP assays by using anti-STAT5a/b antibody. Genomic/protein precipitants were PCR-amplified by using primers against the E1 site (CTTCCCTTCTTCAGGGTGCT/TCACGCCTCCTGTTCTTTCT) or the E2 site (TAGCTCAAGGAGGCAGGTTG/GGGCATGAGTGGAAAAGAGA) sites (Fig. 4A). Sequences of the primers used for HEY1 ChIP assays against human PROX1 are as follows (Primer Set 1: GAGAGGCTCGGTCCCACT/TGAGTAATGGGAGGCTCTTTTC; Primer Set 2: GAGCCTCCCATTACTCAGACC/GAGGCTCCCGCTTAGAAACT). EMSA was performed as previously described [22], [54]. Briefly, nuclear lysates isolated from BECs or LECs transduced with a control or IL3Rα-expressing adenovirus were incubated with ^32^P-labeld oligonucleotide probes containing the putative E1 site for STAT5a/b (ATCTGGTTGTAATTCTCAGAATTGGTT/TCCTAAACAAACCAATTCTGAGAATTA) or the E2 site (GCTTGTTTTTATTTTTCCGAGAAGATC/GACAGCACAGATCTTCTCGGAAAAATA) in the binding buffer and subjected to polyacrylamide gel electrophoresis. For EMSA blocking assays, the nuclear extracts were added to the binding reactions in the presence of a STAT5a/b antibody (1 µg/ml).

### Luciferase assay

The reporter constructs of PROX1 promoter were constructed as follows. A 5.1-kb human PROX1 promoter was PCR-amplified from human genomic DNA using primers (GTCCAGGGCGTGTACTGAG/CGGCTGCAATGGTGTATTATT) and cloned into *EcoR*V site of pBluescriptIISK(-) in a reverse orientation. A *Nhe*I/*Xho*I fragment was then transferred to *Nhe*I/*Xho*I sites of pGL3 (Promega) to generate the PROX1_P1 vector. Subsequently, a 1.6-kb *Pst*I or a 3.3-kb *Mlu*I fragment was deleted from PROX1_P1 and self-ligated after Klenow treatment to construct PROX1_P2 and PROX1_P3, respectively. Luciferase reporter constructs [55] for mouse HEY1, HEY2 and HEYL were generously provided by Dr. Eric Olson (University of Texas Southwestern Medical Center, Dallas). The reporter constructs of the mouse Hey1 promoter were constructed by modifying a 2.9-kb mouse Hey1 construct [56] (renamed here as pHey1A) generously provided by Dr. Manfred Gessler (Theodor-Boveri-Institut fuer Biowissenschaften, Germany). Deletion constructs were generated by self-ligation after digesting pHey1A with *EcoR*V/*Msc*I (pHey1B), *EcoR*V/*Apa*I (pHey1C), *EcoR*V/*Asc*I (pHey1D), *EcoR*V/*Sac*II (pHey1E) or *EcoR*V/*Nco*I (pHey1F). Luciferase assays were performed as follows. HEK293T was transiently transfected in 12-well plates in DMEM, 10% FCS. Cells were transfected with a total of 1 ug of DNA by Lipofectamin 2000 (Invitrogen). After 48 hours, cells were washed and lysed in 200 µL of PBS by three-time-repetition of freeze-thaw cycles. Cells were harvested and cell debris was removed by centrifugation. Protein concentrations were measured by Bradford assay and luciferase activity was measured using the Bright-Glo buffer (Promega) by Mikrowin2000 program, Luminometer (Plate CHAMELE, HIDEX). Each experiment was repeated 3 times with each reaction measured in triplicates.

## Supporting Information

Figure S1 **Identification of the putative Stat5 binding sites in the PROX1 promoter regions in nine mammals.** A multiple genome alignment tool, MAUVE [57], was used to align the PROX1 upstream sequences (∼12-kb upstream from the translation initiation codon) from human, chimp, mouse, rat, guinea pig, horse, rabbit, dog and marmoset. PROX1 translation initiation sites (Prox1 ATG codon) and relative locations (box) of the putative Stat5 binding sites of each species are marked in the genomic maps. Cross-species sequence conservations are estimated by red peaks and the asterisks mark the genomic areas where DNA sequence is unavailable. Genome assemblies used for this DNA sequence alignment are as follows: Human (Feb. 2009, GRCh37/hg19), Chimp (Mar. 2006, CGSC 2.1/panTro2), Mouse (July 2007, NCBI37/mm9), Rat (Nov. 2004, Baylor 3.4/rn4), Guinea pig (Feb. 2008, Broad/cavPor3), Horse (Sep. 2007, Broad/equCab2), Rabbit (Apr. 2009, Broad/oryCun2), Dog (May 2005, Broad/canFam2), Marmoset (March 2009, WUGSC 3.2/calJac3). Actual DNA sequences of the putative Stat5 binding sites are shown in the bottom table.
(PDF)

Figure S2 **Effect of Notch activation in human primary LECs.** Adenoviral expression of NICD in primary LECs resulted in suppression of LEC-phenotypes, including downregulation of PROX1, PDPN and CDK1NC.
(PDF)
