# Correction: Opposing Regulation of PROX1 by Interleukin-3 Receptor and NOTCH Directs Differential Host Cell Fate Reprogramming by Kaposi Sarcoma Herpes Virus

**DOI:** 10.1371/journal.ppat.1004544

**Published:** 2014-11-06

**Authors:** The PLOS Pathogens Staff

The authors would like to correct an error in panel A of Figure 1. Some of the western blot panels depicted in Figure 1A were switched with those in Figure 1B during assembly of the final figure. The authors have replaced the Western blots in Figure 1A with a set of panels from an independent experiment ran under the same conditions. Please see the corrected version of Figure 1 here.

**Figure 1:**
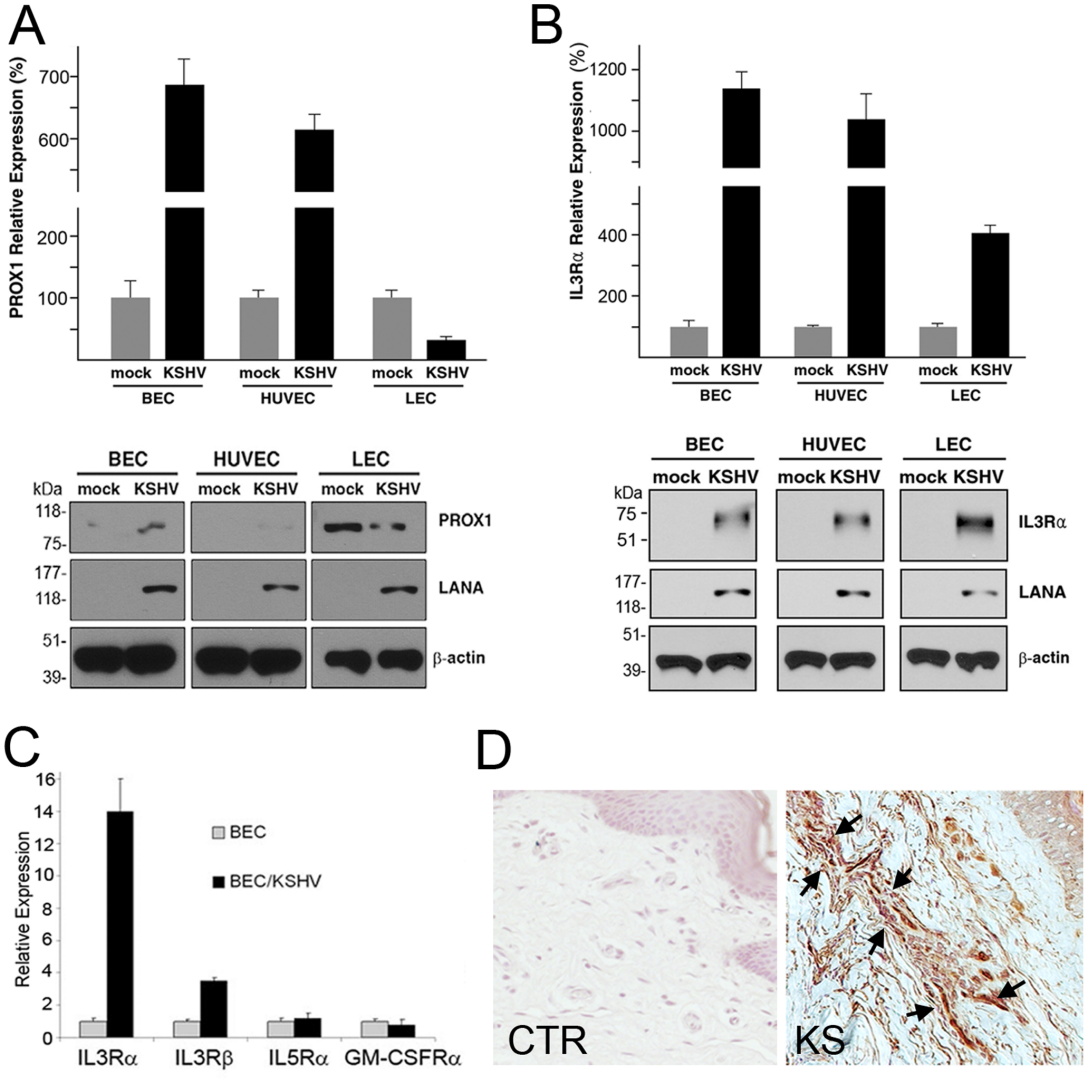
Regulation of the expression of PROX1 and IL3Rα by KSHV in blood vs. lymphatic-lineage endothelial cells. (A) KSHV upregulated PROX1 in BECs and HUVECs, but downregulated PROX1 in LECs based on quantitative real-time RT-PCR (qRT-PCR) and western blot analyses. Latency-associated nuclear antigen (LANA) was used to confirm KSHV-infection and β-actin for equal loading in western analyses. (B) IL3Rα was comparably upregulated by KSHV in the three cell types determined by qRT-PCR and western blot analyses. (C) Expression of IL3Rβ, IL5Rα and GM-CSFRα in BECs by KSHV infection was determined by qRT-PCR. (D) Immunohistochemistry analysis showed prominent expression of IL3Rα in KS tumor cells (arrow-marked) in the skin of a HIV-positive patient. CTR, a control skin section from a normal neonatal foreskin; KS, Kaposi sarcoma tumor section from a HIV-positive individual.
