# Supplementary material for: Opposing Regulation of PROX1 by Interleukin-3 Receptor and NOTCH Directs Differential Host Cell Fate Reprogramming by Kaposi Sarcoma Herpes Virus
Source: PLoS Pathog. 2012 Jun 14;8(6):e1002770. doi: 10.1371/journal.ppat.1002770 (PMC3375311; doi:10.1371/journal.ppat.1002770)
Supplement: Figure S1 — Identification of the putative Stat5 binding sites in the PROX1 promoter regions in nine mammals. A multiple genome alignment tool, MAUVE [57], was used to align the PROX1 upstream sequences (∼12-kb upstream from the translation initiation codon) from human, chimp, mouse, rat, guinea pig, horse, rabbit, dog and marmoset. PROX1 translation initiation sites (Prox1 ATG codon) and relative locations (box) of the putative Stat5 binding sites of each species are marked in the genomic maps. Cross-species sequence conservations are estimated by red peaks and the asterisks mark the genomic areas where DNA sequence is unavailable. Genome assemblies used for this DNA sequence alignment are as follows: Human (Feb. 2009, GRCh37/hg19), Chimp (Mar. 2006, CGSC 2.1/panTro2), Mouse (July 2007, NCBI37/mm9), Rat (Nov. 2004, Baylor 3.4/rn4), Guinea pig (Feb. 2008, Broad/cavPor3), Horse (Sep. 2007, Broad/equCab2), Rabbit (Apr. 2009, Broad/oryCun2), Dog (May 2005, Broad/canFam2), Marmoset (March 2009, WUGSC 3.2/calJac3). Actual DNA sequences of the putative Stat5 binding sites are shown in the bottom table. (PDF) [file ppat.1002770.s001.pdf]

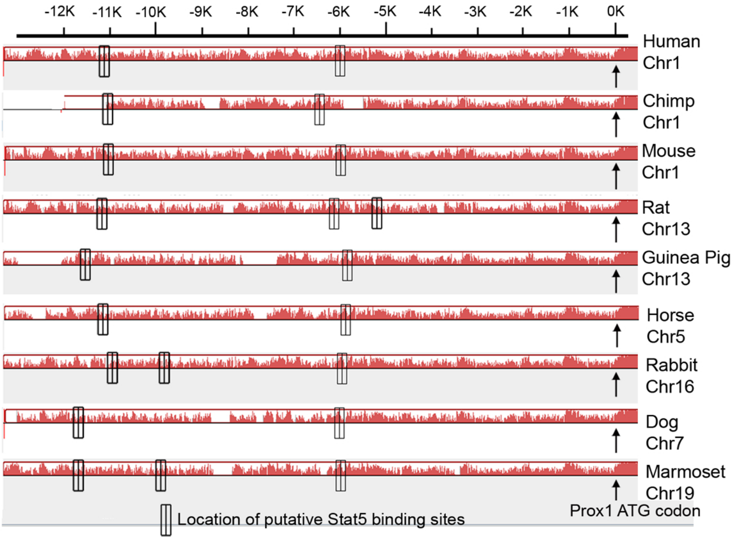

Species	Consensus Sequence (TTCYNRGAA)
Human	TTCTCAGAA, TTCCGAGAA
Chimp	TTCTATGAA, TTCCGAGAA
Mouse	TGCTGGGAA, TTCCTAGAA
Rat	TTTTAAGAA, TTCCTAGAA, TTCTCAGAA
Guinea Pig	TTCTTGGAG, TTCCGAGAA
Horse	TTCCCAGAA, TTCCAAGAA
Rabbit	TTCCCGGAA, TTCTCAGAA, TTCCAAGAA
Dog	TTCCCAGAA, TTCCAAGAA
Marmoset	TTCCCAGAA, TTCCTAGAA, TTCCGAGAA

Supplemental Fig.S1
