# Supplementary figures and images for: Opposing Regulation of PROX1 by Interleukin-3 Receptor and NOTCH Directs Differential Host Cell Fate Reprogramming by Kaposi Sarcoma Herpes Virus
Source: PLoS Pathog. 2012 Jun 14;8(6):e1002770. doi: 10.1371/journal.ppat.1002770 (PMC3375311; doi:10.1371/journal.ppat.1002770)

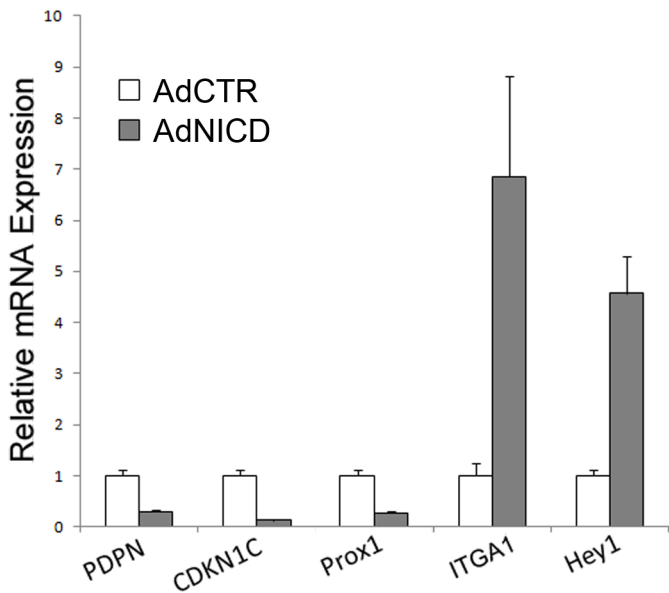

Supplemental Fig.2

Supplement: Figure S2 — Effect of Notch activation in human primary LECs. Adenoviral expression of NICD in primary LECs resulted in suppression of LEC-phenotypes, including downregulation of PROX1, PDPN and CDK1NC. (PDF) [file ppat.1002770.s002.pdf]
